# Shorter total sleep time is associated with lower CD4+/CD8+ T cell ratios in virally suppressed men with HIV

**DOI:** 10.1093/sleepadvances/zpae001

**Published:** 2024-01-17

**Authors:** Priya V Borker, Bernard J Macatangay, Joseph B Margolick, Naresh M Punjabi, Charles R Rinaldo, Valentina Stosor, Joshua Hyong-Jin Cho, Heather McKay, Sanjay R Patel

**Affiliations:** Division of Pulmonary Allergy, Critical Care and Sleep Medicine, University of Pittsburgh, Pittsburgh, PAUSA; Division of Infectious Diseases, University of Pittsburgh, Pittsburgh, PAUSA; Department of Molecular Microbiology and Immunology, Johns Hopkins Bloomberg School of Public Health, Baltimore, MD, USA; Division of Pulmonary, Critical Care, and Sleep Medicine, University of Miami Miller School of Medicine, Miami, FL, USA; Division of Infectious Diseases, University of Pittsburgh, Pittsburgh, PAUSA; Divisions of Infectious Diseases and Organ Transplantation, Northwestern University, Chicago, IL, USA; Cousins Center for Psychoneuroimmunology, Department of Psychiatry and Biobehavioral Sciences, University of California Los Angeles, Los Angeles, CAUSA; Department of Epidemiology, Johns Hopkins Bloomberg School of Public Health, Baltimore, MD, USA; Division of Pulmonary Allergy, Critical Care and Sleep Medicine, University of Pittsburgh, Pittsburgh, PAUSA

**Keywords:** total sleep time, HIV, T lymphocytes

## Abstract

**Study Objectives:**

Although poor sleep quality is associated with lower CD4+ T cell counts among people living with HIV (PLWH), the association between objective sleep metrics and T lymphocyte subset counts is unknown. We evaluated the association between polysomnography (PSG) derived sleep metrics and T lymphocyte subpopulations in a cohort of men living with HIV.

**Methods:**

Virally suppressed men living with HIV participating in the Multicenter AIDS Cohort Study underwent home overnight PSG. We assessed the association of PSG parameters with CD4+ and CD8+ T cell counts and the CD4+/CD8+ T cell ratio.

**Results:**

Overall, 289 men with mean (±SD) age 55.3 ± 11.3 years and mean CD4+ T cell count 730 ± 308 cells/mm^3^ were evaluated. Total sleep time (TST) was significantly associated with CD8+ but not CD4+ T cell counts. After adjusting for age, race, depressive symptoms, antidepressant use, and non-nucleoside reverse transcriptase inhibitors use, every hour of shorter TST was associated with an additional 33 circulating CD8+ T cells/mm^3^ (*p* = 0.05) and a 5.6% (*p* = 0.0007) decline in CD4+/CD8+ T cell ratio. In adjusted models, every hour of shorter rapid eye movement (REM) sleep was associated with an additional 113 CD8+ T cells/mm^3^ (*p* = 0.02) and a 15.1% lower CD4+/CD8+ T cell ratio (*p* = 0.006). In contrast, measures of sleep efficiency and sleep-disordered breathing were not associated with differences in T lymphocyte subpopulations.

**Conclusions:**

Our findings suggest that shorter TST and REM sleep durations are associated with differences in T lymphocyte subpopulations among men living with HIV. Addressing sleep may reflect a novel opportunity to improve immune function in PLWH.

**Statement of Significance**

The significance of sleep health on immune function in people living with HIV (PLWH) is poorly understood. Deviations in circulating T lymphocyte subpopulations, such as depressed CD4+ T lymphocyte counts, expanded CD8+ T lymphocyte counts, or low CD4+/CD8+ T cell ratios, can be signs of impaired immune function or heightened immune activation in PLWH. We investigated the association between polysomnography-derived sleep metrics and T lymphocyte subsets in a cohort of virally suppressed men living with HIV. We found that shorter total sleep time, and particularly shorter REM sleep duration, was associated with greater CD8+ T lymphocyte counts and lower CD4+/CD8+ T cell ratios in PLWH. These results suggest that poor sleep may contribute to differences in T lymphocyte subpopulations and chronic immune activation in PLWH.

## Introduction

Sleep disturbances are common in people living with HIV (PLWH), with a prevalence as high as 30%–80% [1–6]. PLWH frequently experiences deficiencies in multiple sleep health dimensions as evidenced by high rates of short sleep duration, insomnia, and obstructive sleep apnea (OSA) [5–12]. In the era of antiretroviral therapy (ART), clinical management of PLWH has transitioned to managing HIV as a chronic condition, with an emphasis on improving quality of life and preventing comorbidities not associated with acquired immunodeficiency syndrome (AIDS). Sleep disturbances among PLWH have been associated with a wide range of adverse clinical consequences, including poor medication adherence, HIV disease progression, and non-AIDS comorbidities such as depression and cardiovascular disease [3, 13–19]. Therefore, understanding how sleep health affects PLWH is of clinical consequence.

Heightened immune activation is linked to important comorbidities and premature mortality among PLWH, and sleep disturbances are associated with impairments in immune function that may contribute to HIV-associated inflammation and chronic immune activation [20–22]. Indeed, sleep disturbances, including short sleep duration, insomnia, and OSA, are independent risk factors for non-AIDS comorbidities common in PLWH [23, 24]. The ratio of CD4+ T lymphocytes to CD8+ T lymphocytes (CD4+/CD8+ ratio) is an immunologic measure associated with increased risk of non-AIDS comorbidities among PLWH. Despite effective ART, PLWH often demonstrates altered T cell subset proportions and function [25]. Lower CD4+/CD8+ T lymphocyte ratios are associated with greater immune activation and immunosenescence and confer a greater risk for the development of comorbidities and mortality among PLWH [26–31].

Sleep disturbances are associated with an altered hematologic profile, including derangements in lymphocyte counts [32–34]. For example, the proportion of CD4+ T lymphocytes increases after exposure to total sleep deprivation and rapid eye movement (REM) sleep deprivation [35]. In another example, absolute CD4+ and CD8+ T lymphocyte counts were lower in persons without HIV with chronic insomnia than compared to a group of “good sleepers” [36]. However, prior research investigating an association between sleep exposure and CD4+ T lymphocyte counts in PLWH has been conflicting and limited primarily to self-reported sleep measures [37, 38]. One study that included objective measures of sleep enrolled PLWH with varying levels of HIV control, including active AIDS, which raises concerns of reverse causality [6]. Research investigating an association between sleep exposure and CD8+ T lymphocyte counts is more sparse and also limited to self-reported sleep measures [39]. We sought to evaluate the association between objective measures of sleep, measured using polysomnography (PSG), and lymphocyte subsets in a cohort of men living with virally suppressed HIV infection.

## Materials and Methods

### Study population

The Multicenter AIDS Cohort Study (MACS) is a longitudinal cohort study evaluating the treatment trajectory and associated comorbidities of men living with HIV [40]. Men having sex with men, both with or and without HIV were enrolled from sites in Baltimore MD/Washington DC, Chicago IL, Pittsburgh PA/Columbus, OH, and Los Angeles, CA in four waves of enrollment (1984–1985, 1987–1991, 2001–2003, and 2010–2017) [40, 41]. The study was approved by the Institutional Review Boards at each site and all participants provided informed consent. In brief, MACS participants attended semiannual study visits where they underwent standardized physical exams, completed standardized interviews, and provided blood samples for virologic, serologic, immunologic, and laboratory measurements, including evaluating HIV status, plasma HIV-1 RNA concentration, and measurements of T cell subset counts [40]. The cumulative duration of HIV infection and antiretroviral therapy (ART) was updated at each visit. The following analyses were restricted to participants with HIV.

### Sleep assessments

From March 2018 to June 2019, MACS participants were eligible to enroll in an ancillary sleep study to evaluate sleep health via home polysomnography (PSG). The protocol for this ancillary study was approved by the institutional review board at each site, and informed consent was obtained from each individual. Participants completed one night of home-based self-applied PSG with a type II portable sleep monitor (Nox A1 PSG; Nox Medical, Reykjavik, Iceland) [42]. The home PSG collected four frontal electroencephalogram (EEG) channels (AF4, AF3, AF7, and AF8), frontalis muscle electromyogram (EMG), left and right electrooculograms (EOG), electrocardiogram (ECG), right and left anterior tibialis electromyogram (EMG), pulse oximetry, nasal airflow, and respiratory inductance plethysmography. After completion, participants returned the sleep monitor to the local study site, where data were downloaded and transmitted to a central reading facility for manual scoring.

Full details on the PSG protocol and the scoring procedures implemented by the central reading facility, which generated the data analyzed here, have been published [42]. Briefly, sleep studies were manually scored for sleep staging using standard criteria [43]. Staging for wakefulness, non-rapid eye movement (NREM) stages N1, N2, N3, and REM sleep was conducted using 30-second epochs. The frontalis muscle EMG was used to identify changes in muscle tone for scoring REM sleep in lieu of chin EMG. Arousals were identified by standard criteria [43]. Time in epochs scored for each stage of sleep was used to calculate the duration of each sleep stage. The total time across all stages was used to define total sleep time (TST). In addition to TST, the following PSG metrics were extracted for analysis: sleep latency (time between the start of the recording to the first epoch scored as sleep), sleep efficiency (the proportion of epochs scored as sleep between the first and last epochs scored as sleep), wake after sleep onset (the total amount of time in epochs scored as wake between the first and last epochs scored as sleep), and arousal index (the number of arousals per hour of TST) [42].

Metrics of sleep-disordered breathing were also extracted by the central reading facility as described [5, 42]. Apneas were scored if airflow was absent or nearly absent for at least 10 seconds. Hypopneas were scored if there was a reduction of 30% or more in airflow for at least 10 seconds with an associated 4% decrease in oxygen saturation. The apnea–hypopnea index (AHI_4_) was defined as the number of apneas and hypopneas per hour of sleep. Additionally, the severity of nocturnal hypoxemia was assessed by the oxygen desaturation index, defined as the number of desaturation episodes of 3% or more during sleep and the percent of time spent in sleep with saturation <90% (TST_90_). High interscorer reliability was observed for TST (inter-rater intra-class correlation coefficient ICC: 0.96), sleep latency (ICC: 0.95), NREM staging (ICC: 0.94), REM staging (ICC: 0.95), arousal index (ICC 0.89), and AHI_4_ (ICC 0.99) [42].

### T lymphocyte assessments

T lymphocyte subset levels were measured semiannually as part of each MACS visit [44]. Blood samples were collected into ethylenediaminetetraacetic acid (EDTA) anticoagulant-containing vacutainer tubes for complete blood count and lymphocyte immunophenotyping. complete blood count assessments were performed with automated hematology analyzers by CLIA-certified clinical reference laboratories. Timing of blood draws was not standardized but most occurred during daytime hours. A detailed procedure has been previously published [44, 45]. Quality control measures to obtain comparable lymphocyte subset measurements at the four MACS study sites have been previously reported, including standardized sample preparation, monoclonal antibody staining, and analytic procedures between study sites [44].

### Covariates

Participants’ self-reported sociodemographic characteristics such as age and race/ethnicity (non-Hispanic white/non-Hispanic black/other) were collected at each MACS semiannual visit. Body mass index (BMI) was calculated from the height and weight measured at the last MACS semiannual visit before the MACS sleep visit. During MACS visits, participants reported all prescribed medications [46], including antidepressant use, which can suppress REM sleep, and ART regimen [47]. We separately coded use of non-nucleoside reverse transcriptase inhibitors because this class of medications, particularly efavirenz, has been associated with effects on sleep [48]. Participants also completed the 20-item Center for the Epidemiologic Studies Depression Scale (CES-D) scale. They were coded as having depressive symptoms if their most recent CES-D score before the sleep exam was 16 or greater, as depression has been associated with increased REM sleep and with differences in the CD4+/CD8+ T cell ratio [46, 49, 50].

### Statistical analyses

Analyses were restricted to participants who had an undetectable viral load (i.e. less than the lower limit of quantification for the assay used) on the most recent viral load before the sleep study and on all assays performed in the year prior to the PSG. Participants were excluded if they were on supplemental oxygen, had an incomplete or poor-quality PSG [42], or if T lymphocyte subset counts were not available within the 365 days prior to the PSG.

The following three measurements were examined as outcomes of interest: CD4+ T-cell count, CD8+ T-cell count, and CD4+/CD8+ T-cell ratio. Associations were evaluated using linear regression models. Lymphocyte subset counts were modeled continuously, but because of the non-normal distribution of the CD4+/CD8+ T cell ratio, this outcome was log-transformed. PSG measures were modeled continuously in primary analyses and categorically in secondary analyses to assess potential non-linear effects. For TST, the categories were defined a priori as short (<6 hours), intermediate (6–7 hours), and long (>7 hours) as a recent meta-analysis suggests that 6–7 hours of sleep duration predicts the lowest mortality [51]. For duration in each sleep stage, categories were defined by tertiles. Secondary models included adjustment for age, race, BMI, depressive symptoms, antidepressant use, and NNRTI use [48, 52]. All analyses were conducted in SAS 9.3 (SAS Institute, Cary, NC).

## Results

### Study population characteristics

**Figure 1.**
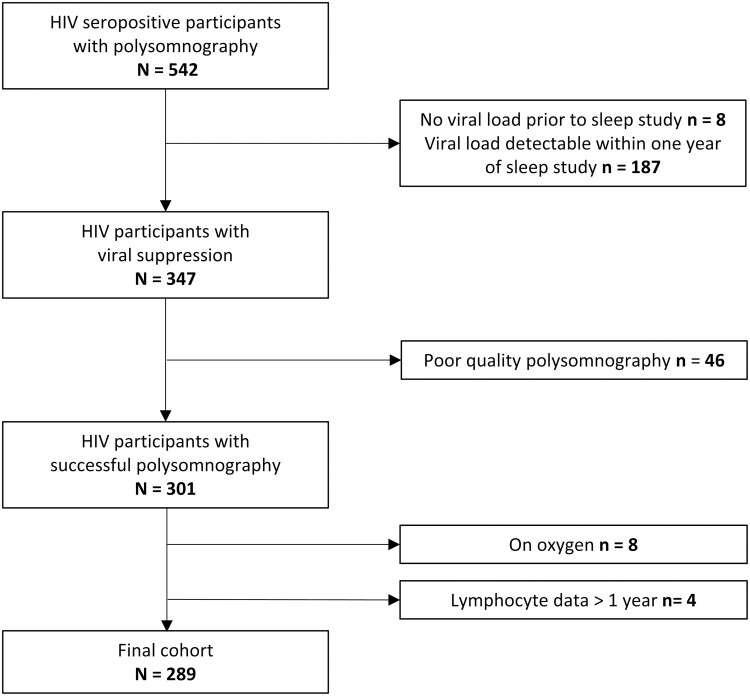
Sample flow chart.

Details regarding study participant inclusion are summarized in Figure 1. Of the 542 PLWH with PSG data, 187 participants were excluded due to evidence of viremia within 1 year prior to the sleep study and eight lacked viral load data before the study. Of the 347 who met the criteria for viral suppression, 46 had poor-quality PSG. Of the remaining 301 participants with valid PSG data, eight participants were excluded due to use of supplemental oxygen, and four had no T lymphocyte subset data within 1 year prior to the study, yielding a total analytic sample of 289 men. Multivariable analyses were limited to a sample of 288 men due to a missing CES-D score in one participant.

Table 1 summarizes the demographic and clinical characteristics of the cohort. Overall, the cohort consisted primarily of white, middle-aged men with mean age of 55.3 ± 11.3 years. Three-quarters (78.9%) were on ART for at least 2 years prior to the PSG. The median time between blood draw and PSG was 1 day (IQR [0–13 days]) and 94% of participants had T lymphocyte counts drawn within 90 days of the sleep study. As shown in Table 2, the mean TST was 6.2 ± 1.4 hours, and 41.9% of the cohort had a TST < 6 hours. Roughly half the cohort (52.3%) had an AHI_4_ ≥ 5 events/h and about one-fifth (19.4%) had an AHI_4_ ≥ 15 events/h.

**Table 1.** Participant Characteristics

	Frequency (%) or Mean ± SD *N* = 289
Age (y)	55.3 ± 11.3
Race/ethnicity	
Non-Hispanic white (*n*,%)	160 (55.4%)
Non-Hispanic black (*n*,%)	83 (28.7%)
Other (*n*,%)	46 (15.9%)
Body mass index (kg/m^2^)	27.2 ± 5.1
CD4+ T-lymphocyte count (cells/mm^3^)	730 ± 308
CD8+ T-lymphocyte count (cells/mm^3^)	814 ± 394
CD4+/CD8+ T cell ratio	1.02 ± 0.54
History of AIDS diagnosis	21 (7.3%)
Antiretroviral therapy duration (y)	3.3 ± 1.8
Antiretroviral therapy	
No ART	3 (1.0%)
Protease inhibitors	52 (18.0%)
Non-nucleoside reverse transcriptase inhibitors	78 (27.0%)
Integrase inhibitors	143 (49.5%)
Other	13 (4.5%)
CES-D score*	10.9 ± 10.6
CES-D ≥ 16*	82 (28.5%)
Antidepressant use	81 (28.0%)

Data are presented as mean ± SD or *N* (%). AIDS, acquired immunodeficiency syndrome; CES-D, center for the epidemiologic studies depression.

^*^CES-D score was available in 288 participants.

**Table 2.** Polysomnography Characteristics of Virally Suppressed Men Living With HIV

Sleep characteristic	Median (IQR)
Total sleep time (h)	6.2 (5.3, 7.0)
Sleep efficiency (%)	93 (85, 96)
Sleep latency (min)	13 (5, 27)
Wake after sleep onset (min)	35 (18, 62)
*Sleep stages*	
Total N1 duration (min)	65.0 (39.5, 92.0)
Percentage of N1 sleep	17.3 (11.6, 33.6)
Total N2 duration (min)	227.5 (187.0, 277.0)
Percentage of N2	63.2 (54.3, 70.9)
Total N3 duration (min)	3.0 (0.0, 21.5)
Percentage of N3 sleep	0.8 (0.0, 6.0)
Total REM sleep duration (min)	50.5 (30.0, 72.0)
Percentage of REM	14.0 (9.4, 18.6)
Sleep-disordered breathing metrics	
Apnea–hypopnea index 4% (events/h)	5.6 (1.9, 11.8)
Oxygen desaturation index 4% (events/h)	5.2 (1.8, 11.6)
Percent of total sleep time spent with oxygen saturation <90% (%)	1.6 (0.1, 9.9)

Data are presented as median and interquartile range from a single night of home-based self-applied polysomnography recording using the Nox A1 system (Nox Medical).

#### T lymphocyte cell count by sleep characteristics.

**Figure 2.**
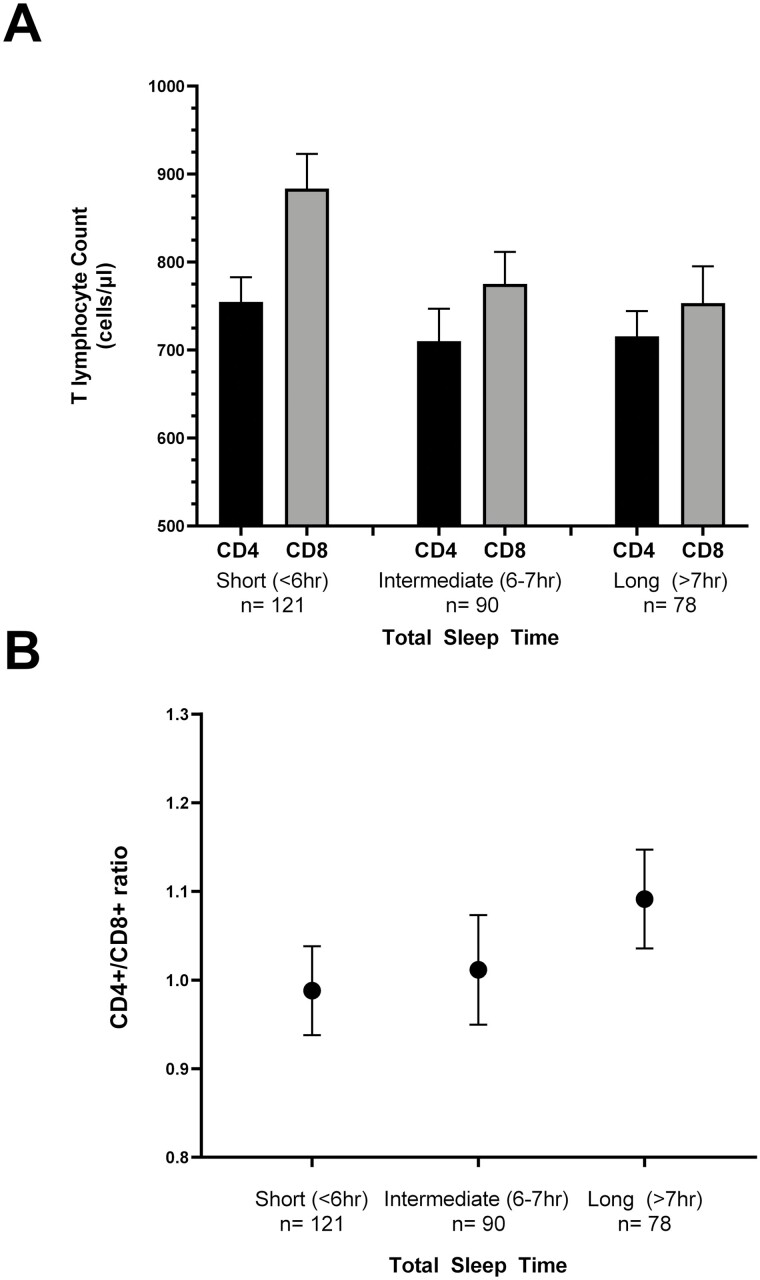
CD4+ and CD8+ T lymphocyte counts and CD4+/CD8+ ratio by sleep duration. (A) Mean CD4+ and CD8+ T lymphocyte count by total sleep time category. Error bars display standard error. (B) Mean CD4+/CD8+ T lymphocyte ratio by total sleep time category. Error bars display standard error.

Although TST was not significantly associated with CD4+ T lymphocyte counts, it was associated with CD8+ T lymphocyte counts. When modeled continuously, every hour decrease in TST was associated with 39 cells/mm^3^ greater CD8+ T lymphocyte counts (*p* = 0.02) (Table 3). This association remained after adjustment for age, race, BMI, depressive symptoms, antidepressant use, and NNRTI use (an additional 33 cells/mm^3^ for each hour of lower TST, *p* = 0.05). Because of the potential U-shaped association between sleep duration and adverse health outcomes, we conducted a sensitivity analysis excluding the six participants with TST ≥ 9 hours, finding no appreciable difference in findings (Supplementary Table S1). When TST was modeled as a categorical variable, no evidence of a U-shaped association was found (Figure 2A). Instead, the mean CD8+ T cell count increased monotonically as TST declined, with the greatest difference observed between those with intermediate and short TST. The CD4+/CD8+ T cell ratio also varied significantly with TST. Every hour decrement in TST was associated with a 5.2% lower CD4+/CD8+ T cell ratio in unadjusted analyses (*p* = 0.01) and a 5.6% lower CD4+/CD8+ T cell ratio (*p* = 0.007) in the adjusted model. In categorical analyses, a monotonic relationship was again observed such that means in CD4+/CD8+ T cell ratios were lower in groups with lower TST (Figure 2B).

**Table 3.** Association Between Total Sleep Time and T Lymphocyte Subsets in Virally Suppressed Men Living With HIV

	CD4+ count (cells/mm^3^)			CD8+ count (cells/mm^3^)			CD4+/CD8+ ratio		
Total sleep time (per hour decrease)	*β*	95% CI	*P*-value	*β*	95% CI	*P*-value	*β*	95% CI	*P*-value
Unadjusted model	6.2	(−19.6, 31.9)	0.64	38.9	(6.3, 71.6)	0.02	−5.2%	(−9.0, −1.2%)	0.01
Adjusted model^*^	1.2	(−24.0, 26.3)	0.93	31.1	(0.8. 67.5)	0.05	−5.6%	(−9.4, −1.6%)	0.007

Results of linear regression modeling T lymphocyte populations and the log-transformed CD4+/CD8+ T lymphocyte ratio by total sleep time on home polysomnography (*N* = 289). The values reported are the effects for each additional hour decrease in total sleep time.

^*^Adjusted for age, race, body mass index, depressive symptoms, antidepressant use, and non-nucleoside reverse transcriptase inhibitor use (*N* = 288).

**Figure 3.**
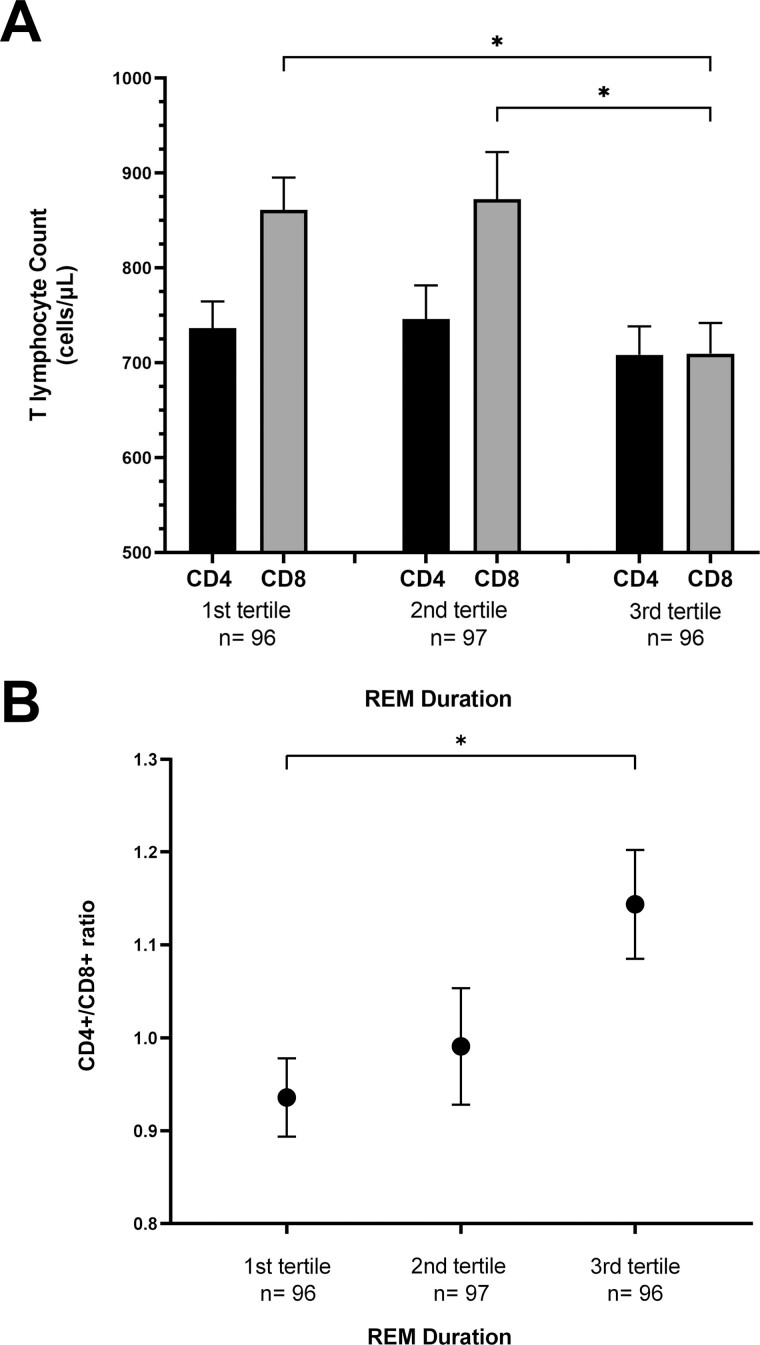
CD4+ and CD8+ T lymphocyte counts and CD4+/CD8+ ratio by REM duration. (A) Mean CD4+ and CD8+ T lymphocyte count by REM duration tertile. Error bars display standard error. first tertile: 0–36.0 minutes, second tertile: 36.5–62.5 minutes, and third tertile: 63.0–159.5 minutes. * denotes *p* < 0.05. (B) Mean CD4+/CD8+ T lymphocyte ratio by REM duration tertile. Error bars display standard error. first tertile: 0–36.0 minutes, second tertile: 36.5–62.5 minutes, and third tertile: 63.0–159.5 minutes. * denotes *p* < 0.05.

To explore whether a particular sleep stage drove the associations between TST and T lymphocyte subset counts, we evaluated the associations between sleep stage durations and T lymphocyte subsets. As with the relationship with TST, we found that CD4+ T cell counts did not vary by the time spent in any sleep stage (Table 4). In contrast, although CD8+ T cell counts were not significantly associated with any NREM sleep stage, an association was found with time spent in REM sleep. CD8+ T lymphocyte counts were 120 cells/mm^3^ greater (*p* = 0.01) for every hour decrement in REM sleep in the unadjusted analysis and 113 cells/mm^3^ greater (*p* = 0.02) in the adjusted analysis. In categorical analyses, the mean CD8+ T cell counts were similar between the bottom and middle tertile but were significantly lower in the top tertile, corresponding to a REM duration ≥ 63.0 minutes (Figure 3A). Similarly, the CD4+/CD8+ T lymphocyte ratio was 15.8% lower (*p* = 0.004) for every hour of REM sleep loss in the unadjusted analysis, and 15.1% lower for every hour of REM sleep loss (*p* = 0.006) in the adjusted analysis. When modeled by tertiles, greater REM duration was associated with greater CD4+/CD8+ T lymphocyte ratios, with the largest difference observed between the intermediate and long REM duration (Figure 3B).

**Table 4.** Association Between Sleep Stage and T Lymphocyte Subsets in Virally Suppressed Men Living With HIV

	CD4+ count (cells/mm^3^)			CD8+ count (cells/mm^3^)			CD4+/CD8+ ratio		
	*β*	95% CI	*P*-value	*β*	95% CI	*P*-value	*β*	95% CI	*P*-value
N1 sleep duration (per hour decrease)	−28.7	(−80.9, 23.5)	0.28	39.2	(−30.5, 108.9)	0.27	−6.5%	(−14.3, 2.0%)	0.13
N2 sleep duration (per hour decrease)	1.2	(28.7, −31.2)	0.94	12.0	(−27.9, 52.0)	0.56	−3.5%	(−8.1, 1.5%)	0.17
N3 sleep duration (per hour decrease)	53.2	(−37.0, 143.4)	0.25	15.4	(−105.3, 136.1)	0.80	3.9%	(−10.6, 20.9%)	0.62
REM sleep duration (per hour decrease)	11.5	(−59.2, 82.3)	0.75	113.3	(19.8, 206.9)	0.02	−15.8%	(−25.1, −5.4%)	0.006

REM, rapid eye movement sleep.

Results of multivariable linear regression modeling T lymphocyte populations by sleep stage duration (*N* = 288). The values reported are the effects for each additional hour decrease in time spent in a particular stage of sleep. All models include adjustment for age, race, body mass index, depressive symptoms, antidepressant use, and non-nucleoside reverse transcriptase inhibitor use.

In additional analyses, we explored the association of T lymphocyte subset populations with PSG-derived insomnia-related and sleep-disordered breathing metrics (Table 5). Longer sleep latency was associated with greater CD8+ T lymphocyte counts (*p* = 0.03) but not with CD4+/CD8+ ratio. In general, no consistent associations were found between T lymphocyte subsets and measures of poor sleep efficiency, sleep fragmentation, or sleep-disordered breathing severity.

**Table 5.** Association Between Insomnia and Sleep Apnea Measures With T Lymphocyte Subsets in Virally Suppressed Men Living With HIV

	CD4+ T lymphocyte count (cells/mm^3^)			CD8+ T lymphocyte count (cells/mm^3^)			CD4+/CD8+ ratio		
	*β*	95% CI	*P*-value	*β*	95% CI	*P*-value	*β*	95% CI	*P*-value
Sleep latency (per 10 minutes)	5.2	(−7.6, 18.0)	0.43	18.9	(1.9, 35.8)	0.03	−1.2%	(−3.3, 0.9%)	0.27
Wake after sleep onset (per 10 minutes)	4.3	(−3.5, 12.2)	0.28	1.1	(−9.4, 11.6)	0.83	0.2%	(−1.1, 1.6%)	0.71
Sleep efficiency (%) (per 10 minutes)	−11.1	(−46.5, 24.3)	0.54	−16.4	(−63.8, 30.9)	0.50	2.3%	(−3.5, 8.6%)	0.44
Arousal index (per 10 arousals/h)	26.1	(−11.6, 63.9)	0.18	4.0	(−46.5, 54.6)	0.88	0.5%	(−5.6, 7.1%)	0.87
Apnea–hypopnea index 4% (per 10 events/h)	4.6	(−24.7, 34.0)	0.76	23.5	(−15.6, 62.6)	0.24	−1.5	(−6.2, 3.4%)	0.54
Oxygen desaturation index 4% (per 10 events/h)	10.6	(−13.1, 34.3)	0.38	26.6	(−5.0, 58.1)	0.10	−1.6	(−5.4, 2.4%)	0.43
Percent of total sleep time spent with oxygen saturation <90% (per 10% total sleep time)	−14.4	(−33.4, 4.7)	0.14	−19.7	(−45.1, 5.8)	0.13	0.7%	(−2.5, 4.0%)	0.67

Results of multivariable linear regression modeling of T lymphocyte populations as a function of sleep measures (*N* = 288). All models include adjustment for age, race, body mass index, depressive symptoms, antidepressant use, and non-nucleoside reverse transcriptase inhibitor use.

## Discussion

Overall, we found that short TST on home polysomnography was associated with a greater number of circulating CD8+ T lymphocytes and a lower CD4+/CD8+ T lymphocyte ratio among virally suppressed PLWH. The associations between CD8+ T lymphocyte and the CD4+/CD8+ T cell ratio and TST were similar between unadjusted models and models adjusting for age, race, depressive symptoms, use of antidepressants, and non-nucleoside reverse transcriptase inhibitors. Differences in T lymphocyte subsets appeared to be driven to a large extent by time spent in REM sleep and persisted despite adjusting for both depressive symptoms and antidepressant use. Sleep latency was associated with differences in CD8+ T cell counts; however, this relationship was not robust as differences in the CD4+/CD8+ T cell ratio were not seen and other measures of insomnia did not appear to be related to T lymphocyte counts. Metrics associated with sleep-disordered breathing severity were not associated with either CD4+ or CD8+ T lymphocyte counts.

PLWH are at higher risk of developing chronic conditions associated with poor sleep health, including cardiovascular disease, malignancies, and neuropsychiatric conditions, compared to the general population [53]. A leading hypothesis is that persistent chronic immune activation leading to premature immunosenescence may play a key role in developing non-AIDS-associated comorbidities [54, 55]. HIV infection drives an expansion of differentiated CD8+ T cell populations, which exhibit characteristics of T cell exhaustion and limited functional capacity. Although CD4+ T lymphocyte counts in many PLWH normalize with ART, quantitative and functional defects in CD8+ T cells may remain even after effective long-term therapy [25, 56]. In particular, a low (<1) CD4+/CD8+ ratio despite peripheral CD4+ T-cell restoration may reflect underlying immune activation and immunosenescence. A low CD4+/CD8+ ratio in the absence of detectable HIV viremia is associated with increased CD4+ and CD8+ T cell activation and CD8+ T cell senescence [57–59]. Importantly, these alterations in immune function associated with a low CD4+/CD8+ ratio contribute to the development of several comorbidities associated with aging [25–29, 60–63]. Similarly, elevated CD8+ T cells are associated with increased risk of incident cardiovascular disease [57–59].

Sleep disturbances are prevalent among PLWH and emerging evidence suggests that poor sleep is a risk factor for developing similar non-AIDS-associated comorbidities [5, 6, 10]. For example, one study found that PLWH who complained of frequent difficulty falling or staying asleep were more than 50% more likely to develop incident cardiovascular disease than those without sleep complaints [17]. As sleep disturbances are associated with functional and phenotypic changes to circulating immune cells in people without HIV [36], it is imperative to investigate if poor sleep health is associated with perturbations to immune function in PLWH. Our study found that every hour reduction in TST is associated with ~30 cells/mm^3^ greater CD8+ T cell count and every hour reduction in REM sleep is associated with ~120 cells/mm^3^ greater CD8+ T cell count, suggesting poor sleep may contribute to differences in circulating CD8+ T cell concentrations and contribute to a lower CD4+/CD8+ T cell ratio in PLWH [64].

There are a number of plausible biological mechanisms by which short sleep duration, and specifically short REM sleep duration, may cause elevations in CD8+ T cell counts and a depressed CD4+/CD8+ ratio. First, the frequency and reactivity of both CD4+ and CD8+ T lymphocytes demonstrate a circadian variation, with both subpopulations demonstrating similar proportional variations throughout the 24-hour period [65]. This diurnal variation is dampened in response to sleep deprivation, suggesting that there is a contributing sleep-related process that contributes to T lymphocyte cycling [32, 33, 65]. Similarly, both short-term and long-term REM sleep deprivation depress circulating T lymphocytes in rats [66]. It is postulated that sleep contributes to immunologic memory by affecting lymphocyte homing and redistribution [67, 68]. Indeed, it has been shown in sheep that lymph flow and efferent lymphocyte output are reduced during sleep compared to wakefulness, directly affecting lymphocyte circulation [69]. Sleep-associated regulation of immune cells is likely mediated via hormone regulation [67, 68]. Sleep-regulated hormones such as prolactin, growth hormone, and aldosterone affect T cell function and localization, including homing to lymph nodes [70–75]. In addition, changes in cortisol caused by sleep deprivation may also impact T lymphocyte homing [70, 76]. The contribution of REM sleep specifically to T cell homing has been less well studied. However, REM sleep is characterized by increased sympathetic output, which impacts a broad range of CD8+ T cell functions such as differentiation, lymph node homing, and survival [77–81]. Independent of its impact on hormonal regulation, emerging evidence demonstrates that both total and REM sleep deprivation induce apoptosis in the gut epithelium, disrupting gut integrity [82, 83]. This phenomenon may be particularly important in HIV infection where gut dysfunction and subsequent microbial translocation are important pathways of chronic immune activation [84, 85].

This study adds to the literature examining the association between CD4+ and CD8+ T lymphocyte counts and sleep health. There have been inconsistent results regarding the association between sleep quality and CD4+ T lymphocytes. Three studies found that PLWH with lower CD4+ T lymphocyte counts were more likely to have poor sleep quality, as measured by the global PSQI score [38, 86, 87]. In contrast, one study found that greater PSQI scores were positively associated with CD4+ T lymphocyte counts [37]. Two additional studies found no association with PSQI score, although both found PLWH with insomnia had greater CD4+ T lymphocyte counts [6, 88]. Research on the association between sleep and CD8+ T lymphocyte counts is sparse. Cruess et al. found that PLWH who reported greater levels of psychological distress and sleep disturbance were more likely to have lower CD8+ T lymphocytes [39]. However, no objective assessments of sleep were made. As the PSQI score is a global composite measure of sleep quality, the inconsistent results may be due to varying effects of different dimensions of sleep health on T lymphocyte counts. Lee et al investigated the association between lymphocyte counts and actigraphy-derived sleep metrics and found that sleep fragmentation but not sleep duration was associated with CD4+ T cell counts [6]. However, this population included a high proportion of participants experiencing active AIDS, poverty, and unemployment, which may confound associations between sleep health and T lymphocyte counts.

Our study has limitations. The cross-sectional design precludes the ability to make temporal or causal inferences about relationships between specific sleep measures and T lymphocyte subsets. Indeed, lymphocyte subset data could not always be acquired on the same day as the PSG. Nevertheless, lymphocyte data were collected within 24 hours in the majority of participants and within 2 weeks in 90% of participants. Future research should also control for the timing of T lymphocyte sampling to reduce variation due to circadian rhythmicity in circulating T lymphocyte counts. Another limitation of our work is that measurement of TST was based on a single night recording of PSG which may not reflect habitual sleep duration. Additionally, while our assessment of sleep used the gold standard of PSG done in the home to minimize first-night effect, the monitoring may have led to disruptions that altered sleep relative to habitual patterns. However, prior research demonstrates that TST measured by home sleep studies well approximates TST measured by actigraphy and is more accurate than self-reported sleep duration [89]. Furthermore, it is difficult to contemplate how disruptions caused by self-applied polysomnogram would be associated with T cell subsets suggesting that any resulting bias is null and that true associations may be stronger than what we report. Nevertheless, future studies utilizing longer-term monitoring to assess habitual sleep patterns, such as with actigraphy, would be helpful in confirming our results. Although all sleep stages were observed, participants in the study demonstrated a low proportion of N3 sleep, which is due to the limitations of using the self-applied forehead EEG signals that have higher impedances leading to attenuated voltages of observed delta waves [90]. Thus, the limited EEG utilized may have affected the study’s power to evaluate differences in this sleep stage on T lymphocyte subsets. Similarly, this cohort did not have significant hypoxemic exposure, which has been associated with cardiovascular risk and early mortality in OSA [91]. It should also be noted that our cohort was limited to men living with HIV and results may not be generalizable to women living with HIV. Similarly, the relevance of our findings to people without HIV is unclear. A recent study suggests that gender modifies the effect of sleep disturbances on immune function [92]. A lower CD4+/CD8+ ratio has been shown to be a poor prognostic factor in the elderly and other disease states [93, 94]. We focused on PLWH as the impact of immune disruption is most relevant to health outcomes in an immunosuppressed population. Nevertheless, further research on the impact of sleep disruption on T lymphocytes in a non-HIV population is warranted.

Differences in T lymphocyte counts may also be due to other nonspecific factors, such as the presence of comorbidities such as diabetes, health behaviors, or environmental differences, that could potentially confound the relationships reported. However, we found the strength of association between TST and CD8+ T lymphocyte counts was minimally impacted by adjustment for depressive symptoms or antidepressant use. Given the increasing evidence that short sleep duration plays a causal role in the development of other chronic diseases such as diabetes and heart disease, we did not adjust for these comorbidities in order not to over-adjust. Nevertheless, we cannot exclude the possibility of residual confounding influencing our findings. Lastly, short sleep duration may be a manifestation of psychological distress or an outcome of social environments, which induces changes in stress hormones or health behaviors, such as adherence to ART, that could lead to poorer HIV control [52, 95–97]. However, the population included in our analyses were limited to virally suppressed men in order to ensure high levels of ART adherence.

Importantly, our results are limited to differences between absolute T lymphocyte counts and the CD4+/CD8+ T cell ratio. While differences in these measures among PLWH have been associated with differences in immune activation and immunosenescence, we did not directly measure markers of immune activation or immunosenescence. Future studies measuring direct markers of immune activation such as CD38 and HLADR and markers of immunosenescence such as CD127 and CD57 will increase understanding of the relationship between sleep and immunity among PLWH.

Our study investigated the association between PSG-derived sleep measures, including measures of sleep architecture and CD4+ and CD8+ T lymphocyte subsets in PLWH from a large, well-characterized cohort study of virally suppressed men with HIV similar in sociodemographic and other risk factors, many of whom have been followed for more than 25 years with in-depth and standardized data collection, specimen collection, processing, and storage. We included participants enrolled across multiple sites, increasing the generalizability of our findings. Furthermore, to best isolate the effect of sleep on the CD4+/CD8+ T lymphocyte ratio, we focused our analyses on PLWH with viral suppression to avoid any confounding effect of viremia or nonadherence with ART.

In summary, we found that among virally suppressed PLWH, a shorter TST was associated with higher CD8+ T lymphocyte counts and lower CD4+/CD8+ ratios. These results suggest that poor sleep may contribute to chronic immune activation and immunosenescence in PLWH on ART. Further research should assess if short sleep duration is associated with greater alterations in CD8+ T cell phenotype from naïve toward higher frequencies of activated, senescent T cells, activated/exhausted T cells, and measures of inflammation. Further research should also explore if sleep-targeted interventions, such as sleep extension, normalize immune alterations in PLWH with sleep disturbances, which may contribute to improved clinical outcomes.

## Supplementary Material

zpae001_suppl_Supplementary_Tables_S1-S2

## Notes

All affiliated authors have reviewed and approved the following manuscript. Each author has contributed to the development of this manuscript.
