# Supplementary material for: Shorter total sleep time is associated with lower CD4+/CD8+ T cell ratios in virally suppressed men with HIV
Source: Sleep Adv. 2024 Jan 17;5(1):zpae001. doi: 10.1093/sleepadvances/zpae001 (PMC10901437; doi:10.1093/sleepadvances/zpae001)
Supplement: zpae001_suppl_Supplementary_Tables_S1-S2 [file zpae001_suppl_supplementary_tables_s1-s2.docx]

# **Sensitivity Analyses**

**Table S1 Association between total slesep time and T lymphocyte subsets in virally suppressed men living with HIV among participants with total sleep duration < 9 hours.**

|  | **CD4+ count**  **(cells/mm^3^)** | | | **CD8+ count**  **(cells/mm^3^)** | | | **CD4+/CD8+ ratio** | | |
| --- | --- | --- | --- | --- | --- | --- | --- | --- | --- |
| Total sleep time  (per hour decrease) | β | 95% CI | p-value | β | 95% CI | p-value | β | 95% CI | p-value |
| Unadjusted Model | 4.5 | (-23.0, 32.0) | 0.75 | 37.9 | (3.0, 72.8) | 0.03 | -5.3% | (-9.4, -1.0%) | 0.02 |
| Adjusted Model* | -5.7 | (-32.5, 21.1) | 0.68 | 31.4 | (-4.3, 67.1) | 0.09 | -6.1% | (-10.2. -1.9%) | 0.006 |

Results of linear regression modeling T lymphocyte populations and the log-transformed CD4+/CD8+ T lymphocyte ratio by total sleep time on home polysomnography among participants with a total sleep duration less than nine hours (N=283). The values reported are the effects for each additional hour decrease in total sleep time.

*Adjusted for age, race, body mass index, depressive symptoms, antidepressant use, and non-nucleoside reverse transcriptase inhibitor use (N=282).

**Table S2: Association between sleep stage and T lymphocyte subsets in virally suppressed men living with HIV with total sleep duration < 9 hours.**

|  | **CD4+ count**  **(cells/mm^3^)** | | | **CD8+ count**  **(cells/mm^3^)** | | | **CD4+/CD8+ ratio** | | |
| --- | --- | --- | --- | --- | --- | --- | --- | --- | --- |
|  | β | 95% CI | p-value | β | 95% CI | p-value | β | 95% CI | p-value |
| N1 Sleep Duration  (per hour decrease) | -36.0 | (-88.9, 17.0) | 0.18 | 33.8 | (-37.1, 104.8) | 0.35 | -6.6% | (-14.6, 2.1%) | 0.13 |
| N2 Sleep Duration  (per hour decrease) | -3.7 | (-35.9, 28.5) | 0.82 | 9.8 | (-33.3, 52.8) | 0.66 | -3.9% | (-9.0, 1.4%) | 0.15 |
| N3 Sleep Duration  (per hour decrease) | 49.6 | (-42.0, 141.2) | 0.29 | 13.7 | (-109.1, 136.6) | 0.83 | 3.4% | (-11.4, 20.6%) | 0.67 |
| REM Sleep Duration (per hour decrease) | 1.7 | (-70.6, 74.1) | 0.96 | 100.0 | (4.0, 196.1) | 0.04 | -14.7% | (-24.4, -3.8%) | <0.01 |

REM: rapid eye movement sleep

Results of multivariable linear regression modeling T lymphocyte populations by sleep stage duration among participants with a total sleep duration less than nine hours (N=282). The values reported are the effects for each additional hour decrease in time spent in a particular stage of sleep. All models include adjustment for age, race, body mass index, depressive symptoms, antidepressant use, and non-nucleoside reverse transcriptase inhibitor use.
